# Retraction: Case–control study to develop and validate a questionnaire for the secondary prevention of endometriosis

**DOI:** 10.1371/journal.pone.0348638

**Published:** 2026-05-05

**Authors:** The PLOS One Editors

The *PLOS One* Editors retract this article [1] because it was identified as one of a series of submissions for which we have concerns about compromised peer review.

All authors did not agree with the retraction.
